# Glucagon-Like Peptide-1 Receptor Agonists and Cardiovascular Events: A Meta-Analysis of Randomized Clinical Trials

**DOI:** 10.1155/2011/215764

**Published:** 2011-04-26

**Authors:** Matteo Monami, Francesco Cremasco, Caterina Lamanna, Claudia Colombi, Carla Maria Desideri, Iacopo Iacomelli, Niccolò Marchionni, Edoardo Mannucci

**Affiliations:** ^1^Section of Geriatric Cardiology and Medicine, Department of Cardiovascular Medicine, University of Florence and Careggi Teaching Hospital, 50141 Florence, Italy; ^2^Diabetes Agency, University of Florence and Careggi Teaching Hospital, 50141 Florence, Italy; ^3^Section of Geriatric Cardiology and Medicine, Department of Cardiovascular Medicine, Azienda Ospedaliero-Universitaria Careggi, Pieraccini Avenue 18, 50141 Florence, Italy

## Abstract

*Objective*. Data from randomized clinical trials with metabolic outcomes can be used to address concerns about potential issues of cardiovascular safety for newer drugs for type 2 diabetes. This meta-analysis was designed to assess cardiovascular safety of GLP-1 receptor agonists. *Design and Methods*. MEDLINE, Embase, and Cochrane databases were searched for randomized trials of GLP-1 receptor agonists (versus placebo or other comparators) with a duration ≥12 weeks, performed in type 2 diabetic patients. Mantel-Haenszel odds ratio with 95% confidence interval (MH-OR) was calculated for major cardiovascular events (MACE), on an intention-to-treat basis, excluding trials with zero events. *Results*. Out of 36 trials, 20 reported at least one MACE. The MH-OR for all GLP-1 receptor agonists was 0.74 (0.50–1.08), *P* = .12 (0.85 (0.50–1.45), *P* = .55, and 0.69 (0.40–1.22), *P* = .20, for exenatide and liraglutide, resp.). Corresponding figures for placebo-controlled and active comparator studies were 0.46 (0.25–0.83), *P* = .009, and 1.05 (0.63–1.76), *P* = .84, respectively. *Conclusions*. To date, results of randomized trials do not suggest any detrimental effect of GLP-1 receptor agonists on cardiovascular events. Specifically designed longer-term trials are needed to verify the possibility of a beneficial effect.

## 1. Introduction

Cardiovascular safety is a growing concern for drugs used for chronic conditions, such as diabetes. Among glucose-lowering agents, sulfonylureas [1, 2], insulin [3, 4], and thiazolidinediones [5–7], have been suspected of adverse cardiovascular effects, although some of those preoccupations have not been confirmed [8–11]. Following these concerns, the Food and Drug Administration issued a guidance for companies submitting new chemical entities as treatments for type 2 diabetes, requiring that, either in phase II-III trials, or in a subsequent phase IV specifically designed randomized clinical trial, a sufficient amount of information is collected so as to exclude a risk increase of over 30% (i.e., the upper limit—two-sided—of 95% confidence interval for major cardiovascular events, in comparison with placebo and/or other treatments, should not exceed 1.30; http://www.fda.gov/downloads/Drugs/GuidanceComplianceRegulatoryInformation/Guidances/UCM071627.pdf).

Two GLP-1 receptor agonists (exenatide and liraglutide) have been approved for human use, and several others are currently under clinical development. It has been observed that chronic stimulation of GLP-1 receptors could produce beneficial effects on several cardiovascular risk factors [12]; furthermore, preliminary data on humans suggest that GLP-1 could have direct effects on myocardial function [13]. However, no major trial assessing the effects of GLP-1 receptor agonists on cardiovascular morbidity and mortality is available to date, nor will it be for a few years. In the meantime, the information on incident cases recorded as adverse events during trials designed for metabolic endpoints could provide some hints on the possible cardiovascular profile of these drugs. This meta-analysis was designed to assess the effect of GLP-1 receptor agonists, compared with placebo or active hypoglycemic drugs, on major cardiovascular events in type 2 diabetic patients, as derived from randomized controlled trials.

## 2. Research Design and Methods

### 2.1. Data Sources and Searches

An extensive Medline, Embase, and Cochrane database search for “exenatide,” “liraglutide,” “albiglutide,” “taspoglutide,” “lixisenatide,” and “semaglutide” was performed, collecting all randomized clinical trials on humans up to November 1th, 2010. The identification of relevant abstracts, the selection of studies based on the criteria described above, and the subsequent data extraction were performed independently by two of the authors (E. Mannucci and M. Monami), and conflicts resolved by the third investigator (N. Marchionni). Completed but still unpublished trials were identified through a search of http://www.clinicaltrials.gov/ website. Food and Drug Administration (FDA, http://www.fda.gov/) and European Medicines Agency (EMEA, http://www.ema.europa.eu/) reviews of approved drugs, as well as published information provided to FDA in response to queries during the approval process, were also searched for retrieval of unpublished trials. 

### 2.2. Study Selection

A meta-analysis was performed including all randomized clinical trials with a duration of at least 12 weeks, either with a cross-over or a parallel series design, enrolling patients with type 2 diabetes, comparing glucagon-like peptide-1 (GLP-1) receptor agonists with placebo or active drugs (oral hypoglycemic agents and/or insulin) of other classes. Trials enrolling nondiabetic, or type 1 diabetic, subjects were also excluded. No review protocol was published elsewhere.

### 2.3. Data Extraction and Quality Assessment

Results of unpublished trials (characteristics of patients enrolled, treatments, and major cardiovascular events) were retrieved, if available, on http://www.clinicaltrials.gov/, http://www.novonordisk-trials.com/website/content/trial-results.aspx, http://www.lillytrials.com/results/results.html, or http://www.clinicalstudyresults.org/; Food and Drug Administration (FDA, http://www.fda.gov/) and European Medicines Agency (EMEA, http://www.ema.europa.eu/) reviews of approved drugs, as well as published information provided to FDA in response to queries during the approval process, were also searched for retrieval of unpublished information. All those sources were also used to complete information on results of published trials, when not reported in publications. For all published trials, results reported in papers were used as the primary source of information, when available.

The quality of trials was assessed using some of the parameters proposed by Jadad et al. [14]. The score was not used as a criterion for the selection of trials whereas some items were used only for descriptive purposes. 

### 2.4. Data Synthesis and Analysis

The principal outcome was the effect of GLP-1 receptor agonists, compared with other hypoglycemic agents or placebo, on major cardiovascular events (MACE) as defined in the list provided by FDA for this purpose (http://www.fda.gov/downloads/AdvisoryCommittees/CommitteesMeetingMaterials/Drugs/EndocrinologicandMetabolicDrugsAdvisoryCommittee/UCM148659.pdf), including cardiovascular death, nonfatal myocardial infarction and stroke, and hospitalizations due to acute coronary syndromes and/or heart failure. 

Predefined separate analyses were performed for trials with different GLP-1 receptor agonists, whenever possible.

Mantel-Haenszel odds ratio with 95% confidence interval (MH-OR) was calculated for each of the events defined above, on an intention-to-treat basis, excluding trials with zero events. A random effect model was used because of the impossibility of a reliable assessment of heterogeneity, due to the small number of events in each trial [15]. Publication bias was not assessed, considering that the small number of adverse cardiovascular events in each study was irrelevant for the decision to publish trials with metabolic endpoints. The main expected bias is represented by the fact that the trials included were designed for noncardiovascular (metabolic) endpoint; this means that cardiovascular events were reported only as adverse events, without any systematic screening or predefined diagnostic criteria. The meta-analysis was reported following the PRISMA checklist [16]. All analyses were performed using Comprehensive Meta-analysis Version 2, Biostat (Englewood, NJ, USA) and SPSS 16.0.

This research was performed independently of any funding, as part of the institutional activity of the investigators. 

## 3. Results

**Figure 1:**
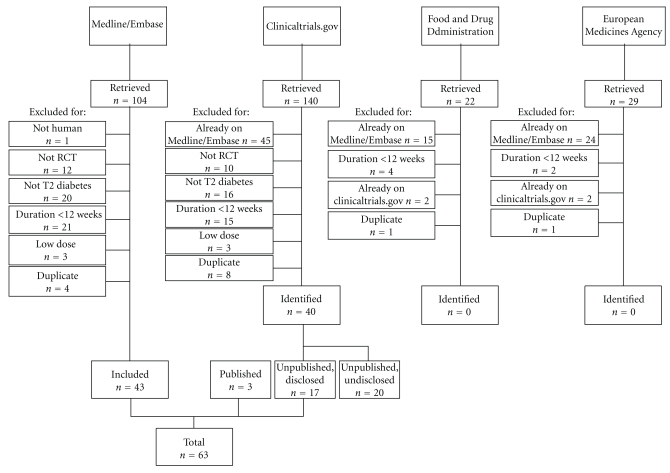
Trial flow diagram. RCT: randomized clinical trial; T2: type 2.

The trial flow is summarized in Figure 1. A total of 36 trials, 3 of which unpublished, were retrieved. Information on major cardiovascular events was reported in 33 trials, 20 of which with at least one event. The analysis on MACE was therefore performed on 20 trials, enrolling 6,490 and 3,995 patients (3.467 and 2.172 patient* years) in the GLP-1 receptor agonist and comparator groups, respectively. The characteristics of the retrieved trials, and of those which resulted to be complete but were undisclosed, or did not report information on MACE, are summarized in Tables 1, 2, and 3.

**Figure 2:**
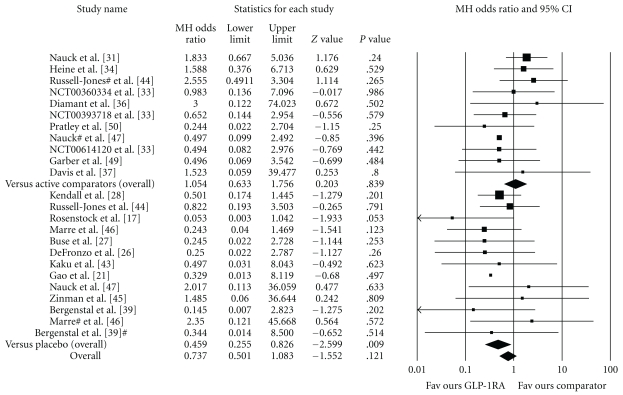
Effect of GLP-1 receptor agonists on fatal and nonfatal major cardiovascular events (MACE). Forest plot of individual studies. GLP-1 RA: glucagon-like peptide-1 receptor agonists. ^#^Studies with multiple comparators.

The total number of patients with events was 65 (0.01%) and 49 (0.01%) in the GLP-1 receptor agonists and comparator groups, respectively. Treatment with the experimental drugs was not associated with an increased incidence of MACE (MH-OR.0.74 (0.50–1.08); *P* = .12). A significant reduction of cardiovascular events with GLP-1 receptor agonists was observed in placebo-controlled trials but not in studies versus active comparators (Figure 2). No consistent pattern suggesting differences between exenatide and liraglutide emerged across analyses. In comparisons with insulin (5 trials with events) and sulfonylureas (4 trials with events), the MH-OR for GLP-1 receptor agonists was 1.77 (0.91–3.44), *P* = .09, and 0.49 (0.22–1.10), *P* = .085, respectively.

All-cause mortality was reported in 33 trials, 9 of which with at least one event (8 and 7) in GLP-1 receptor agonists and comparator, respectively; MH-OR for experimental drugs was 0.67 [0.26–1.78], *P* = .43. 

## 4. Conclusions

The reduction of cardiovascular morbidity and mortality is one of the main aims of long-term treatment of hyperglycemia in type 2 diabetes. Therefore, the possibility of an increased cardiovascular risk associated with some hypoglycemic treatments [1, 3–7] is almost paradoxical. Although some data on adverse cardiovascular effects of specific drugs were not confirmed by subsequent investigations [8–11], the concerns of health authorities about the safety of new compounds appear to be justified (http://www.fda.gov/downloads/Drugs/GuidanceComplianceRegulatoryInformation/Guidances/UCM071627.pdf). In order to reach definitive conclusions on cardiovascular safety of any drug, large-scale, long-term trials should be performed prior to marketing; unfortunately, this effort would be economically unfeasible for pharmaceutical companies. The FDA accepted a compromise, allowing the organization of such trials after drug approval, as a condition for the maintenance of marketing authorization. The limit of this approach is that cardiovascular safety of new drugs will be established only several years after their approval, leaving clinicians without reliable information on this critical point in the meantime. 

Meta-analyses of cardiovascular events recorded as adverse events in randomized clinical trials designed for other purposes can represent an additional source of information. This approach has several limitations, most notably the lack of predefined diagnostic criteria and screening methods for incident cardiovascular disease, with the risk of misdiagnosis and underdiagnosis. It should also be recognized that in some of the trials included, cardiovascular events were reported only as adverse events, without being prospectively adjudicated. Moreover, the limited duration of trials designed for metabolic purposes can impair their ability to detect longer-term effects on atherogenesis. Furthermore, the meta-analysis of small trials with few events each poses some specific, and complex, statistical problems [17]. All these limitations affected the reliability of results of some meta-analyses [6, 7] on cardiovascular safety of hypoglycemic drugs [10, 17, 18].

Those considerations should be taken into account when interpreting the results of the present meta-analysis, which exclude, at least in the short term, any major adverse effect of GLP-1 receptor agonists on cardiovascular morbidity. Interestingly, those drugs, as a class, are below to the 1.3 threshold chosen by the FDA for the upper limit of 95% confidence interval to establish the cardiovascular safety of a new drug. 

Interestingly, a significant reduction of cardiovascular morbidity with GLP-1 receptor agonists was observed in comparison with placebo. This result should be discussed with great caution, considering the limitations highlighted above; in fact, a meta-analysis of trials performed for different (noncardiovascular) endpoints provides reliable information on safety, but not on efficacy. Speculatively, several mechanisms could underlie a beneficial effect of GLP-1 receptor agonists on cardiovascular risk. Reduction of blood glucose, body weight, and blood pressure, as well as favorable effects on lipid profile, have all been reported. Direct myocardial effects of GLP-1 receptor stimulation could theoretically reduce the functional impact of myocardial ischemia [13], leading to clinical improvements. However, the possibility of a beneficial action of GLP-1 receptor agonists on cardiovascular events should be confirmed through specifically designed randomized clinical trials. 

In conclusion, GLP-1 receptor agonists do not appear to increase cardiovascular morbidity in comparison with placebo or other active drugs. Any possible beneficial action should be assessed in further trials. 

##  Author Contributions

M. Monami organized the collection of clinical data, prepared and revised the paper, and performed data analysis. F. Cremasco collected clinical data and assisted in study design and data analysis. C. Lamanna collected clinical data and revised the paper. C. Colombi collected clinical data and assisted in study design. S. Zannoni collected clinical data. I. Iacomelli collected clinical data N. Marchionni reviewed/edited the paper. E. Mannucci designed the study, prepared and revised the paper, and took part in data analysis. 

##  Conflict of Interests

The corresponding author confirms that he had full access to all the data in the study and had final responsibility for the decision to submit for publication. M. Monami has received speaking fees from Eli Lilly and Sanofi-Aventis. F. Cremasco is currently employed by Eli Lilly. N. Marchionni has received speaking fees from Eli Lilly, Novo Nordisk, and Sanofi-Aventis, and research grants from Eli Lilly, Novo Nordisk, and Sanofi-Aventis. E. Mannucci has received consultancy fees from Eli Lilly and Novo Nordisk, speaking fees from Eli Lilly, Novo Nordisk, and Sanofi-Aventis, and research grants from Eli Lilly, Novo Nordisk, and Sanofi-Aventis. 

## Figures and Tables

**Table 1:** Characteristics of the unpublished and undisclosed studies.

Study	Number of patients planned	Comparator	Add-on to	Trial duration (wks)	Design	Study end date*	Sponsor
*Exenatide *							
NCT00434954	488	Aspart	Metformin	26	PS, DB	August 2009	Amylin
*Liraglutide*							
NCT00696657	415	Placebo	None	12	PS, DB	February 2009	Novo
*Taspoglutide*							
NCT00809705	60	Placebo	None	12	PS, DB	February 2010	Hoff-Roche

PS: parallel series; DB: double blind; Hoff. Roche: Hoffman-La Roche; Novo: Novo Nordisk.

**Table 2:** Characteristics of the studies included in the meta-analysis.

Study* (ref.)	NCT/FDA-reference	Add-on to	Description of randomization	Description of allocation	Description of blinding	Reporting of drop-out	Intention-to-treat
*Albiglutide versus placebo*							
Rosenstock et al. [19]	NCT00518115	None/Metf.	NA	NA	A	A	Yes

*Exenatide versus placebo*							
Gill et al. [20]	NCT00516074	Metf./TZD	NA	NA	A	A	Yes
Kadowaki et al. [21]	NCT00382239	Sulfonylurea	A	NA	A	A	Yes
Zinman et al. [22]	NCT00099320	TZD	A	A	A	A	Yes
Gao et al. [23]	NCT00324363	SU + Metf.	A	NA	A	A	Yes
DeFronzo et al. [24]	NCT00135330	Rosiglitazone	A	NA	OL	A	Yes
Apovian et al. [25]	NR	Multiple	A	A	A	A	Yes
Moretto et al. [26]	NCT00381342	None	A	A	A	A	Yes
Liutkus et al. [27]	NR	Metf/TZD + Met	A	A	A	A	Yes
DeFronzo et al. [28]	NCT00039013	Metformin	A	NA	A	A	Yes
Buse et al. [29]	NCT00039026	Sulfonylurea	A	NA	A	A	Yes
Kendall et al. [30]	NCT00035984	SU + Metf.	NA	NA	A	A	Yes
*Exenatide versus rosiglitazone*							
DeFronzo^#^et al. [24]	NCT00135330	None	A	NA	OL	A	Yes
*Exenatide versus glibenclamide*							
Derosa et al. [31]	NCT00135330	None	A	NA	OL	A	Yes
*Exenatide versus BiAsp 30/70*							
Bergenstal et al. [32]	NCT00097877	SU + Metf.	A	A	OL	A	Yes
Nauck et al. [33]	NCT00082407	SU + Metf.	A	A	OL	A	Yes
*Exenatide versus glargine*							
Barnett et al. [34]	NCT00099619	SU + Metf.	A	A	OL	A	Yes
NCT00360334 [35]	NCT00360334	OAD	NR	NR	OL	A	Yes
Heine et al. [36]	NCT00082381	SU + Metf.	A	A	OL	A	Yes
Bunck et al. [37]	NCT00097500	Metformin	A	NA	OL	A	Yes
Diamant et al. [38]	NCT00641056	SU + Metf./Metf	A	A	OL	A	Yes
*Exenatide versus insulin*							
Davis et al. [39]	NCT00099333	SU/Metf.	NA	NA	OL	A	Yes

*Exenatide LAR versus placebo*							
Kim et al. [40]	NCT00103935	Metf./None	A	A	A	A	Yes
*Exenatide LAR versus pioglitazone*							
Bergenstal et al. [41]	NCT00637273	None	A	A	A	A	Yes
*Exenatide LAR versus sitagliptin*							
Bergenstal et al. [41]^#^	NCT00637273	None	A	A	A	A	Yes

*Liraglutide versus placebo*							
Madsbad et al. [42]	FDA_1310	None	NA	NA	A	A	Yes
Vilsbøll [43]	NCT00154401	None	NA	NA	A	A	Yes
Seino et al. [44]	FDA_1334	None	A	A	A	A	Yes
Kaku et al. [45]	NCT00395746	Sulfonylurea	NA	NA	NA	NA	Yes
Russell-Jones et al. [46]	NCT00331851	SU + Metf.	A	A	A	A	Yes
Zinman et al. [47]	NCT00333151	Metf. + TZD	A	A	A	A	Yes
Marre et al. [48]	NCT00318422	Sulfonylurea	NA	NA	A	A	Yes
Nauck et al. [49]	NCT00318461	Metformin	A	A	A	A	Yes
*Liraglutide versus metformin*							
Feinglos et al. [50]	NR	None	NA	NA	NA	A	No

*Liraglutide versus rosiglitazone*							
Marre^#^et al. [48]	NCT00318422	Sulfonylurea	NA	NA	A	A	Yes

*Liraglutide versus glimepiride*							
Madsbad^#^et al. [42]	NR	None	NA	NA	OL	A	Yes
NCT00614120 [35]	NCT00614120	Metformin	NR	NR	OL	NR	NR
Nauck^#^et al. [49]	NCT00318461	Metformin	A	A	OL	A	Yes
Garber et al. [51]	NCT00294723	None	A	A	OL	A	Yes

*Liraglutide versus glibenclamide*							
NCT00393718 [35]	NCT00393718	None	NR	NR	OL	NR	NR

*Liraglutide versus sitagliptin*							
Pratley et al. [52]	NCT00700817	None	A	A	OL	A	Yes

*Liraglutide versus glargine*							
Russell-Jones^#^et al. [46]	NCT00331851	SU + Metf.	A	A	A	A	Yes

*All the studies are multicenter and designed as parallel series, with the exception of NCT00099619 which is a cross-over trial; ^#^studies with multiple comparators. Metf.: metformin; NA: not adequate or not adequately reported; A: adequate; TZD: thiazolidinediones; TZD + Met.: thiazolidinediones + metformin; SU + Metf.: sulfonylureas and metformin; OL: open-label; OAD: oral antidiabetic drugs; NR: not reported; SU/Metf: sulfonylureas or metformin; LAR: long-acting release.

**Table 3:** Moderators and outcome variables in individual studies included in the meta-analysis.

Study (ref.)	Number of patients (ID/C)	Trial duration (wks)	Age (ys)	Duration of DM (ys)	HbA1c/FPG baseline (%/mmol/L)	BMI baseline (Kg/m^2^)	MACE (n,ID/C)	All-cause mortality (n,ID/C)	Cardiovasc. mortality (n,ID/C)
*Albiglutide versus placebo*									
Rosenstock et al. [19]	128/50	16	54	5	8.0/9.7	32.0	0/3	NR/NR	NR/NR

*Exenatide versus placebo*									
Gill et al. [20]	27/25	12	55	NR	7.3/NR	NR	0/0	0/0	0/0
Kadowaki et al. [21]	115/40	12	59	11	8.0/9.1	25.9	0/0	0/0	0/0
Zinman et al. [22]	121/112	16	56	8	7.9/8.9	34.0	0/0	0/0	0/0
Gao et al. [23]	234/232	16	55	8	8.3/9.3	26.2	0/1	0/0	0/0
DeFronzo et al. [24]	47/45	20	56	NR	7.9/NR	NR	0/0	0/0	0/0
Apovian et al. [25]	96/98	24	55	5	7.6/8.6	33.7	0/0	0/0	0/0
Moretto et al. [26]	155/77	24	54	1	7.8/8.7	31.5	0/0	0/0	0/0
Liutkus et al. [27]	111/54	26	54	6	8.2/9.1	33.5	0/0	0/0	0/0
DeFronzo et al. [28]	223/113	30	53	6	8.2/9.4	34.0	1/2	0/0	0/0
Buse et al. [29]	248/123	30	55	6	8.6/10.3	33.5	1/2	0/0	0/0
Kendall et al. [30]	486/247	30	55	9	8.5/9.9	34.0	7/6	0/1	0/1

*Exenatide versus rosiglitazone*									
DeFronzo^#^et al. [24]	45/45	20	56	NR	7.9/NR	NR	0/0	0/0	0/0
*Exenatide versus glibenclamide*									
Derosa et al. [31]	63/65	52	56	NR	8.8/7.9	28.6	NR/NR	0/0	0/0
*Exenatide versus BiAsp 30/70*									
Bergenstal et al. [32]	124/248	24	52	NR	10.1/11.4	33.8	NR/NR	0/1	0/1
Nauck et al. [33]	253/248	52	58	10	8.6/11.1	30.4	10/5	2/1	1/1

*Exenatide versus glargine*									
Barnett et al. [34]	138/138	16	55	7	8.9/12.0	31.3	0/0	0/0	0/0
NCT00360334 [35]	118/116	26	56	NR	8.6/10.8	34.1	2/2	NR/NR	NR/NR
Heine et al. [36]	282/267	26	59	9	8.2/10.2	31.3	5/3	0/0	0/0
Bunck et al. [37]	36/33	52	58	5	7.5/9.1	30.6	NR/NR	NR/NR	NR/NR
Diamant et al. [38]	233/232	26	58	8	8.3/9.8	32.0	1/0	0/0	0/0

*Exenatide versus insulin*									
Davis et al. [39]	33/16	16	53	11	8.1/8.7	34.0	1/0	0/0	0/0

*Exenatide LAR versus placebo*									
Kim et al. [40]	30/14	15	53	4	8.4/10.7	36.0	0/0	0/0	0/0

*Exenatide LAR versus pioglitazone*									
Bergenstal et al. [41]	160/165	26	52	6	8.5/9.1	32.0	0/3	0/0	0/0

*Exenatide LAR versus sitagliptin*									
Bergenstal et al. [41]^#^	160/166	26	52	6	8.5/9.1	32.0	0/1	0/1	0/0

*Liraglutide versus placebo*									
Madsbad et al. [42]	135/29	12	57	4	7.5/NR	30.4	0/0	0/0	0/0
Vilsbøll [43]	123/40	14	56	4	8.3/11.8	30.1	0/0	0/0	0/0
Seino et al. [44]	180/46	14	57	8	8.3/NR	23.9	0/0	0/0	0/0
Kaku et al. [45]	176/88	24	60	10	8.4/NR	24.9	1/1	0/0	0/0
Russell-Jones et al. [46]	232/115	26	57	9	8.3/9.2	30.6	5/1	1/2	0/2
Zinman et al. [47]	355/175	26	55	9	8.5/10.1	33.7	1/0	0/0	0/0
Marre et al. [48]	695/114	26	56	6	8.4/9.7	29.7	3/2	0/0	0/0
Nauck et al. [49]	724/121	26	57	7	8.4/10.0	31.2	6/0	1/0	0/0

*Liraglutide versus metformin*									
Feinglos et al. [50]	176/34	12	53	5	7.0/NR	34,5	0/0	0/0	0/0

*Liraglutide versus rosiglitazone*									
Marre^#^et al. [48]	695/232	26	56	6	8.4/9.7	29.7	3/0	0/0	0/0

*Liraglutide versus glimepiride*									
Madsbad^#^et al. [42]	135/26	12	57	4	7.5/NR	30.4	0/0	0/0	0/0
NCT00614120 [35]	698/231	16	53	7	NR/NR	25.5	3/2	0/0	0/0
Nauck^#^et al. [49]	724/121	26	57	7	8.4/10.0	31.2	6/2	1/0	0/0
Garber et al. [51]	498/248	52	53	5	8.3/9.4	33.0	2/2	0/1	0/0

*Liraglutide versus glibenclamide*									
NCT00393718 [35]	268/132	24	58	8	8.3/NR	24.8	4/3	1/0	0/0

*Liraglutide versus sitagliptin*									
Pratley et al. [52]	446/219	26	55	6	8.4/10.0	32.8	1/1	1/1	0/1

*Liraglutide versus glargine*									
Russell-Jones^#^et al. [46]	232/234	26	57	9	8.3/9.2	30.6	5/1	1/1	0/1

^#^Studies with multiple comparators; DM: diabetes mellitus; FPG: fasting plasma glucose; MACE: major cardiovascular events; cardiovasc.: cardiovascular; NR: not reported.
